# Developing an assessment scale for long-term care reablement literacy in home care workers in Taiwan using a modified Delphi method

**DOI:** 10.1186/s12877-020-01854-8

**Published:** 2020-11-04

**Authors:** Hsiao-Wei Yu, Tzu-Ying Chiu, Pin-Yuan Chen, Tai-Hsiang Liao, Wen-Hui Chang, Mei-Wen Wang, Pay-Shin Lin

**Affiliations:** 1 Department of Gerontological Care and Management, College of Nursing, Chang Gung University of Science and Technology, Taiwan; No.261, Wen-Hua 1st Rd., Gui-Shan Dist., Taoyuan City, Taiwan (R.O.C.); 2 Geriatric and Long-term Care Research Center, Chang Gung University of Science and Technology, Taiwan; No.261, Wen-Hua 1st Rd., Gui-Shan Dist., Taoyuan City, Taiwan (R.O.C.); 3 Healthy Aging Research Center, Chang Gung University, Taiwan; No.259, Wen-Hua 1st Rd., Gui-Shan Dist., Taoyuan City, Taiwan (R.O.C.); 4 Department of Family Medicine, Keelung Chang Gung Memorial Hospital, Taiwan; No. 222, Mai-Jin Rd., An-Le Dist., Keelung City, Taiwan (R.O.C.); 5 Graduate Institute of Long-term Care, College of Nursing, Tzu Chi University of Science and Technology, Taiwan; No. 880, Sec.2, Chien-Kuo Rd., Hualien City, Taiwan (R.O.C.); 6 Social Affairs Bureau, Taichung City Government, Taiwan; No.99, Sec. 3, Taiwan Blvd., Xi-Tun Dist., Taichung City, Taiwan (R.O.C.); 7 Wu Gun Shing Physical Therapy Clinic, Taiwan; No. 297, Zhong-Yang Rd., Xin-Zhuang Dist., New Taipei City, Taiwan (R.O.C.); 8 Chunghwa Senior Care Corporation, Taiwan; No. 71-8-2, Zhou-Zi St., Nei-Hu Dist., Taipei City, Taiwan (R.O.C.); 9 Department of Physical Therapy, College of Medicine, Chang Gung University, Taiwan; No.259, Wen-Hua 1st Rd., Gui-Shan Dist., Taoyuan City, Taiwan (R.O.C.)

**Keywords:** Reablement, Literacy, Home care worker, Taiwan

## Abstract

**Background:**

Reablement is a philosophy of change in long-term care (LTC). Assessing the knowledge and competence of LTC professionals who provide reablement services is vital in LTC research. This study aimed to develop a scale for the assessment of long-term care reablement literacy (LTCRL) and employ this scale to assess the performance of home care workers in Taiwan.

**Methods:**

To develop this scale, we employed the modified Delphi technique based on the theoretical framework of health literacy and the content of service delivery in reablement. Home care workers from northern, central, and southern Taiwan were selected through purposive sampling (*N* = 119). Participants answered a self-administered questionnaire that included items related to basic demographic characteristics and questions to assess LTCRL.

**Results:**

Based on the experts’ consensus on the procedure of the modified Delphi technique, the LTCRL assessment sale consists of 29 questions on four aspects of knowledge acquisition: the abilities to access/obtain, understand, process/appraise, and apply/use. The results revealed that higher education levels and better Chinese language proficiency are associated with higher LTCRL outcomes among home care workers.

**Conclusions:**

The LTCRL assessment scale based on a modified Delphi technique is useful and feasible for evaluating LTCRL in home care workers who provide reablement services in Taiwan.

**Supplementary Information:**

The online version contains supplementary material available at 10.1186/s12877-020-01854-8.

## Background

The aging population in Taiwan has increased rapidly. The percentage of elderly individuals was over 15% in 2019 and is expected to exceed 20% by 2025 [1]. In 2010, a survey on the need for long-term care (LTC) in Taiwan reported that 14.95% of elderly Taiwanese individuals had difficulties performing activities of daily living (ADLs), including instrumental ADLs (IADLs), and/or cognitive impairment [2]. “Disability” is defined as difficulty performing socially defined roles and tasks, such as those involving personal hygiene, including bathing, eating, and toileting (ADLs), as well as those needed for living independently, including performing house chores, using public transportation, and managing money (IADLs) [3, 4]. In studies performed in Taiwan, individuals with disabilities were assessed using standard tools, including the Barthel index for ADLs and Lawton’s scale for IADL disabilities [4, 5]. Because of an increase in the number of aging individuals and an expected increase in the number of elderly individuals with disabilities in Taiwan, in 2016, the government introduced the National 10-Year Long-Term Care Plan Version 2.0, which emphasized home- and community-based services [2]. A recent statistical report showed that compared with that in 2018, the number of individuals using home- and community-based services increased by 56% in the first half of 2019 [6]. The demand for home care services to help elderly people with disabilities will increase considering that most elderly Taiwanese individuals prefer to remain in their own homes as they age [2].

With an increase in the aging population and projected demands for home care services in many countries including Taiwan, reablement may be an option for individuals to receive conventional home care services [7, 8]. Reablement refers to the optimization of users’ ability to perform daily activities through a time-limited, goal-tailored, and interdisciplinary team approach conducted in the home or local community [9]. Reablement aims to help users learn or re-learn functional skills necessary for daily activities instead of having someone else perform them [7]. Studies have shown that compared those who received conventional home care services, those who received reablement as an intervention had better scores on self-care and home management ADLs [10], physical functions [11], and self-reported activity performance and satisfaction [12]. Researchers also reported evidence of long-term cost savings with reablement, mainly because of the reduced use of personal care services [13, 14]. Reablement is considered to be effective, and Taiwan has officially introduced reablement to help elderly individuals re-learn or regain their ability to perform ADLs independently; the use of reablement is also an attempt to reduce LTC-related expenditure in the long term [8].

Compared with conventional home care services, which mainly assist service users to perform ADLs, reablement supports service users in regaining functions through the users’ active participation [9]. Professionals involved in reablement have stated that it is a different way of thinking and acts as a shift in the caring culture to transform elderly individuals from passive recipients to active participants [15]. In Taiwan, physical and occupational therapists supervise reablement and are encouraged to work with home care workers, who are the main workforce that delivers services in the service users’ home [8]. The official training process of home care workers in Taiwan begins with an entry-level program that includes 50 h of lectures and a 40-h internship in LTC institutions. After the entry-level program, eligible home care workers must attain an average of 20 h in-service education per year with special topics for the purpose of advancing their caring skills (a total of 120 h in 6 years) [16]. However, because the Taiwanese reablement guidelines and operating manual were launched in 2019, there has been limited evidence of the service quality of reablement home care workers [8]. Therefore, the knowledge and competency of delivering reablement among home care workers need to be evaluated.

To understand an individual’s process of executing health-related knowledge and competence, researchers could adopt the model of health literacy. The health literacy model is used to measure the degree of an individual’s capacity to obtain, process, and understand basic health information and apply it in improving the service user’s well-being [17]. Based on the conceptual model, the generating process of health literacy requires four competencies: accessing, understanding, appraising, and applying health-related information. “Access” refers to the ability to seek and find information; “understand” refers to the ability to comprehend the information that has been accessed; “appraise” refers to the ability to interpret and evaluate the information; and “apply” refers to the ability to use the information to make decisions for the purpose of maintaining or improving health [17].

Previous studies have shown that a health care worker’s individual characteristics and working experience in the health care context are related to health literacy. An individual’s health literacy is associated with age, gender, and employment status [18–20]. Researchers have suggested that educational attainment and readiness play critical roles in determining health literacy [21]. In recent years, the discussion on health literacy has been moving beyond a focus on the individual and toward a focus on the interaction between healthcare service users and providers. A review article implied that efforts to evaluate literacy-directed practices among health professionals might improve the effectiveness of patients’ health outcomes [22]. Coleman et al. [23] expanded the knowledge and established a set of health literacy practices and educational competencies for health professionals. However, these literacy-directed competencies among health professionals mostly applied to those in the acute care setting. Limited empirical data for examining the general abilities to access, understand, appraise, and apply the information relevant to LTC practices in the home-based setting have been reported.

Because the elderly population is increasing considerably and the demands for home care services have increased in Taiwan, the cultural change in home care services from “doing everything for elderly people (conventional home care)” to “helping elderly people learn or re-learn functional skills necessary to perform daily activities (reablement)” is a way of saving LTC cost [8]. Considering that home care workers are the first-line service providers involved in reablement, their competencies in using their caring knowledge to help the elderly regain daily functions should be developed and evaluated. However, to the best of our knowledge, there remains limited information regarding literacy-directed practices among home care workers involved in reablement in Taiwan. In this study, we incorporated the conceptual framework of health literacy and the practical use of reablement in Taiwan. This study aimed to develop a scale for the assessment of long-term care reablement literacy (LTCRL) and employ this scale to assess the LTCRL performance of home care workers in in Taiwan.

## Methods

Based on the consensus among the expert panel, the modified Delphi method was employed and was followed by two steps of data collection. In this study, the steps of the modified Delphi method consisted of a face-to-face meeting with experts and three rounds of email questionnaires. First, an expert panel prepared survey questionnaires regarding the assessment of reablement literacy for home care workers in Taiwan. Second, we conducted a series of structured questionnaires to achieve consensus on the opinions of respondents. The Delphi method is recommended for use in a healthcare setting given that it is an iterative process that employs repeated rounds of voting and feedback from panels to achieve consensus wherein little or no definitive evidence or measurement tools have been developed [23]. We also conducted a pilot study to test LTCRL among home care workers and to identify the reliability of the LTCRL assessment scale. Briefly, a modified Delphi approach was generated by initial scale item construction, followed by obtaining panel experts’ consensus on the content validation of the scale items. After development of the scale, we performed a pilot test to examine the LTCRL performance of home care workers in Taiwan.

### Construction of the LTCRL assessment scale

In the initial step of the modified Delphi method, we conducted literature reviews and met with experts to clarify the core competencies of home care workers involved in reablement in Taiwan. Based on the reviews and experts’ advice, we determined that the core competencies of reablement included a range of components targeted to optimize service users’ performance of daily activities and functions [7, 8, 24]. These may include functional training to re-learn or regain self-care skills, adapting to the environment and using assistive technology, receiving education about self-management behaviors, and use of local resources. In terms of the structures of question sets of the LTCRL scale, we referred to the European Health Literacy Survey Questionnaire, which consists of several scenario-based questionnaires with multiple-choice answers [25]. Each question was answered as either correct (1) or incorrect (0) by the participants. After drawing conclusions, the principles and structures of the survey questionnaires for the scale of LTCRL were developed.

Because the purpose of the LTCRL scale was to evaluate literacy-directed practices among home care workers involved in reablement, we recruited a group of five experts from four professional fields. This expert panel was composed of two physical therapists, one occupational therapist, one nurse who supervised home care workers, and one researcher who was an expert in public health education. These experts were recommended by five benchmark organizations that provide reablement and home care services in Taiwan, including one government-funded assistive technology center, two non-profit organizations, and two private LTC institutions. In addition, all the experts had experience in reablement, and they worked as consultants for home care workers involved in reablement.

### Obtaining consensus from the Delphi panel experts

Subsequently, an expert panel of 10 LTC and rehabilitative professionals rated LTCRL competencies on a 5-point Likert scale and provided written feedbacks and recommendations. We recruited Delphi experts from different professional fields with snowball sampling. The invitation letters were initially sent to two individuals who were experts in the LTC and reablement policy in Taiwan. These experts were asked to recruit future participants from among their acquaintances. We collected the lists of recommendations and finalized 10 experts for the Delphi panel representing five different fields (rehabilitation, geriatric nursing, caring skills, health promotion, and gerontology). The majority were practitioners who had experience with LTC and/or reablement services (*n* = 6); the rest were LTC policymakers and health care educators (*n* = 4). The respondents were blinded to individual scores but were unblinded to the result of all aggregate scores for each question set in the previous round. If a predetermined threshold for each survey questionnaire was agreed by all experts in terms of the competency and qualification for the question, a consensus was achieved. The predetermined threshold for each survey questionnaire was set as the scores of interquartile range (IQR) ≤1 and median ≥ 4 [26, 27]. We collected the data obtained using the modified Delphi method and achieved expert consensus in March and April 2019 via e-mail and online discussion.

### Pilot testing of the LTCRL assessment scale

In addition to the process of the modified Delphi method, we conducted a pilot assessment to examine the reliability and feasibility of the scale and examined the association of characteristics and LTCRL among home care workers in Taiwan. Moreover, we applied the Kuder–Richardson Formula 20 (K-R-20) to test the internal consistency reliability. We recruited samples by means of an in-service educational agency that offers training courses for home care workers in Taiwan. Our research team attended the workshop on reablement and provided a thorough briefing of the study for the purpose of recruitment. The inclusion criteria were home care workers who (1) had provided home care services for more than 3 months and (2) had basic command of Chinese reading and writing. The exclusion criterion was home care workers who provided services in an LTC institution. The exclusion criterion was necessary because not all the workers provided services at users’ homes although they were registered as home care workers. Some worked in LTC institutions or hospitals. Thus, because we aimed to assess the literacy among care workers involved in reablement in a home-based setting, the workplace was an important criterion of the study. Based on the letters of consent we received at the workshop and the inclusion/exclusion criteria of the study, home care workers from northern, central, and southern Taiwan were selected through purposive sampling (*n* = 119).

The included home care workers answered self-administered questionnaires that consisted of items related to basic demographics, self-rated general Chinese proficiency, the working experiences of being a home care worker, and questions of the LTCRL assessment scale that we had developed using the modified Delphi method. Written informed consent was obtained from all participants or their authorized representative as per the institutional review board (IRB)-approved research protocol. Hard copies of self-administered questionnaires and IRB informed consent were completed by the participants. We examined the correlation between the samples’ characteristics and LTCRL scale scores. Differences in the performance between two groups were assessed using Student *t*-test; for multiple groups, we employed analysis of variance. All statistical tests were two-tailed, and a 5% significance level was maintained throughout the analyses. The analytical process was generated using SAS version 9.4 (SAS Institute Inc., Cary, NC, USA).

## Results

A three-round Delphi method was utilized. Ten respondents from five different professional fields answered the survey, and the response rates for the three-round Delphi method were 100, 100, and 90%. Table 1 shows the demographic characteristics of those on the Delphi panels. Of the 10 respondents, the average age was 41.30 years (standard deviation [SD] = 6.25), and most respondents were female (70.00%) and had master’s or higher degrees (90.00%). More than half of the experts had experience in reablement and/or LTC services (60.00%), and others were LTC policymakers and health care educators (40.00%). The professional fields of those on the Delphi panels included rehabilitation, geriatric nursing, caring skills, health promotion, and gerontology.

**Table 1:** Demographic characteristics of the Delphi expert panel (*n* = 10)

Demographic characteristics of the Delphi panels			
Age (years)	41.30 (6.25)	Years in geriatric or health care practices	5.30 (4.67)
Female	7 (70.00%)	Years in geriatric or health care research and education	3.15 (3.20)
Highest level of education		Experience in reablement service	
Bachelor’s	1 (10.00%)	Yes	6 (60.00%)
Master’s	5 (50.00%)	No	4 (40.00%)
Doctorate	4 (40.00%)	Self-reported professional fields	
		Rehabilitation (Yes)	3 (30.00%)
		Geriatric nursing (Yes)	5 (50.00%)
		Caring skills (Yes)	6 (60.00%)
		Health Promotion (Yes)	7 (70.00%)
		Gerontology (Yes)	4 (40.00%)

Statistics are expressed as mean ± standard deviation (SD) for continuous variables and n (%) for categorical variables

The categories for the competencies of LTCRL included the abilities to access/obtain, understand, process/appraise, and apply/use the health-related information (Table 2). The complete list of categories and competencies with the final Likert score on the three-round Delphi is presented in Supplementary Appendix 1. A total of 33 questions were developed for the initial LTCRL assessment scale. However, four items from the survey questionnaires were not considered in the three rounds of Delphi because these items are not commonly exhibited by home care workers involved in reablement in Taiwan. After three rounds of feedback, four categories consisting of 29 items of survey questionnaires achieved the predetermined threshold consensus scores: median ≥ 4 and IQR ≤ 1 for each item. The number of items in each category was as follows: 5 for access/obtain, 4 for understand, 11 for process/appraise, and 9 for apply/use.

**Table 2:** Category and competency of home care workers in LTCRL

Category	Number of items	Competency in LTCRL
Access/obtain	5	• Ability to access reablement-related information, such as using adoptive equipment or environmental adjustment. • Ability to determine reablement-related stakeholders.
Understand	4	• Ability to understand home exercise or training programs relevant to reablement.
Process/appraise	11	• Ability to interpret and evaluate reablement-related information, such as home exercise and using adoptive equipment. • Ability to identify contraindications and avoid risk factors in social and physical contexts.
Apply/use	9	• Ability to utilize knowledge relevant to reablement and make decisions on adopting skills in social and physical contexts.
Overall	29	

The scores of each LTCRL category are shown in Table 3. To calculate the average score for each category, we divided the sum score by the number of items within each category. We found that the home care workers demonstrated the best performance in the category of understand (0.99 ± 0.05), followed by the categories of access/obtain (0.96 ± 0.09), apply/use (0.86 ± 0.12), and process/appraise (0.79 ± 0.13). Meanwhile, the internal consistency reliability for the scale K-R-20 was 0.53.

**Table 3:** LTCRL scale scores (*N* = 119)

Category	Sum score	Average score (divided by the number of questionnaire items)
Access/obtain (0–5)	4.81 ± 0.47	0.96 ± 0.09
Understand (0–4)	3.95 ± 0.22	0.99 ± 0.05
Process/appraise (0–11)	8.71 ± 1.41	0.79 ± 0.13
Apply/use (0–9)	7.72 ± 1.08	0.86 ± 0.12
Overall score (0–29)	25.19 ± 2.27	0.87 ± 0.08

Statistics are expressed as mean ± SD

Table 4 summarizes the basic information of the participants enrolled for the pilot test. The average age of the participants was 46.92 (SD = 9.73), and most were female (*n* = 98, 82.35%). More than half of them earned a bachelor degree (*n* = 67, 56.30%). Furthermore, 86.55% perceived that they had good general Chinese proficiency, and 60% had provided home care services for more than 1 year. Table 5 presents the correlation between the demographic characteristics and LTCRL among home care workers in Taiwan. Age, gender, and the years of working experience were not significantly correlated with LTCRL performance among Taiwanese home care workers. However, participants with higher educational levels had better overall LTCRL scores (*p* < 0.001), especially in the categories of process/appraise (*p* = 0.011) and apply/use (*p* = 0.003). Additionally, home care workers with better self-rated general Chinese proficiency had a marginal positive effect on the scores of the process/appraise category (*p* = 0.046).

**Table 4:** Basic information of home care workers in the study (*N* = 119)

Characteristics		Characteristics	
**Gender**		**General Chinese proficiency**	
Male	21 (17.65)	Not good	16 (13.45)
Female	98 (82.35)	Good/Fluent	103 (86.55)
**Age (years)**	46.92 ± 9.73	**Experience of being a home care worker (years)**	
**Education Level**		< 0.5	25 (21.01)
Below undergraduate	52 (43.70)	0.5–1	19 (15.97)
Undergraduate and above	67 (56.30)	1–3	29 (24.37)
		3–5	14 (11.76)
		> 5	32 (26.89)

Statistics are expresses as mean ± SD for continuous variables and number (%) for categorical variables

**Table 5:** Correlation between characteristics and LTCRL scores among home care workers (*N* = 119)

Samples’ characteristics	Overall score (0–29)	Categories of LTCRL			
		Access/obtain (0–5)	Understand (0–4)	Process/appraise (0–11)	Apply/use (0–9)
**Gender**					
Male	24.86 ± 2.22	4.71 ± 0.46	3.95 ± 0.22	8.52 ± 1.36	7.67 ± 0.97
Female	25.27 ± 2.28	4.83 ± 0.48	3.95 ± 0.22	8.76 ± 1.42	7.73 ± 1.11
**Age**					
< 47 years	25.56 ± 2.30	4.87 ± 0.34	3.98 ± 0.13	8.80 ± 1.53	7.91 ± 1.04
≥ 47 years	24.88 ± 2.21	4.75 ± 0.56	3.92 ± 0.27	8.64 ± 1.30	7.56 ± 1.10
**Education level**					
Below undergraduate	23.40 ± 2.00**	4.75 ± 0.48	3.92 ± 0.27	8.35 ± 1.20**	7.38 ± 1.12**
Undergraduate and above	**25.81 ± 2.28**	4.85 ± 0.47	3.97 ± 0.17	**9.00 ± 1.50**	**7.99 ± 0.98**
**General Chinese proficiency**					
Not good	24.25 ± 2.27	4.88 ± 0.34	3.94 ± 0.25	8.06 ± 1.24*	7.38 ± 1.15
Good/Fluent	25.34 ± 2.24	4.80 ± 0.49	3.95 ± 0.22	**8.82 ± 1.41**	7.78 ± 1.07
**Experience of being a home care worker (years)**					
< 0.5	25.76 ± 1.71	4.88 ± 0.33	3.96 ± 0.20	9.20 ± 1.26	7.72 ± 1.06
0.5–1	25.26 ± 1.28	4.74 ± 0.45	3.95 ± 0.23	8.58 ± 0.96	8.00 ± 1.05
1–3	25.48 ± 2.53	4.76 ± 0.44	3.97 ± 0.19	8.72 ± 1.62	8.03 ± 0.87
3–5	25.50 ± 2.53	4.86 ± 0.36	4.00 ± 0.00	9.00 ± 1.36	7.64 ± 1.39
> 5	24.31 ± 2.58	4.81 ± 0.64	3.91 ± 0.30	8.28 ± 1.49	7.31 ± 1.06

Statistics are expressed as mean ± standard deviation. * *p* < 0.05, ** *p* < 0.01, *** *p* < 0.001. Student *t*-test and ANOVA were used to identify differences between the LTCRL scores and sample characteristics. We divided samples into two age groups based on the average age of the participants (46.92)

## Discussion

In this study, we used the modified Delphi method to develop the Chinese version of the LTCRL assessment scale. After one expert meeting, three rounds of Delphi process, and a pilot assessment of the LTCRL performance of home care workers in Taiwan, the scale was found to be feasible and reliable in the pilot test. The higher the education levels of home care workers in Taiwan, the better was the LTCRL performance, especially in the process/appraise and apply/use categories.

In the Chinese version of the LTCRL assessment scale, we incorporated both the concept of health literacy and reablement literatures [17, 24] and then modified the scenario-based questionnaires to fit the common scenario of reablement in Taiwan. The scale was completed through a three-round Delphi process by 10 respondents from five professional fields. Latif and colleagues stated that a good result of the Delphi process can be achieved even with a small panel of 10 individuals [26]. We invited a panel of experts who had experiences in reablement, and most of them work as supervisors for reablement in both private and public healthcare settings; thus, the scale met face/content validity. Regarding reliability, the coefficient of K-R-20 was 0.53, which is acceptable (> 0.5) for clinical practice [28]. The reason for the low reliability could be that the proportion of the responses to the items was highly correct. The average score of the scale is 25.19, and the highest score obtainable is 29. The dichotomy of the responses designed for the scale might be the other reason for the low reliability. Nevertheless, after a structured expert consensus was obtained throughout the modified Delphi method and the pilot assessment, the Chinese version of the LTCRL scale appears to be feasible for assessing LTCRL among home care workers in Taiwan.

In our pilot test, we recruited the participants with purposive sampling, and most were willing to join the study. Only 1 of 120 participants declined to join the study (recruitment rate, 99.17%). Throughout the pilot test, none of the participants reported difficulties in reading the question items, and all participants completed the questionnaires within 15 min as expected. Based on the framework of Medical Research Council guidance, any problems that undermine the feasibility of the evaluation process should be addressed [29]. Previous studies have reported that participant recruitment, retention, acceptability, and compliance, as well as the expected sample size for predicting the study outcomes through pilot tests, need to interpret carefully [30]. Although the present study showed it is feasible to assess LTCRL among home care workers in Taiwan, we suggest that future studies should be interpreted carefully, and we present our findings with caution in terms of the feasibility issues.

One of the roles of home care workers is being a personal trainer for elderly individuals who are involved in reablement [31]. Herein, the ability to absorb knowledge as well as apply it to the clinical setting is essential. We found that the Taiwanese home care workers were good at the access/obtain and understand categories but not in the later skillful and advanced competencies of process/appraise and apply/use categories. Furthermore, the education levels were related to the process/appraise and apply/use categories, which is consistent with previous studies in which people with higher education levels performed better on health-related literacy [18, 19, 21]. In addition, our findings shed light on the existing educational program for home care workers in Taiwan. The teaching strategies for the current in-service education in Taiwan are mostly lectures and case reports [16], which are more likely linked to basic literacy (access/obtain and understand). We recommend that the education programs further emphasize on scenario-based exercises related to advanced competencies of literacy. Thus, home care workers involved in reablement would have better skills in evaluating caring problems and in making optimal decisions to help the elderly re-learn or regain their ability to perform ADLs independently.

According to this study, the roles of education and self-rated general Chinese proficiency were important in LTCRL. Clearly, individuals with higher education levels would perform better and better utilize knowledge for practical use than those with lower education levels. This finding is consistent with results in the literature, which showed that education had a positive effect on health-related literacy [18, 19, 21]. Surprisingly, the working experience of home care workers was not significantly associated with literacy in this study, possibly because we measured the variable by the number of working years and not the status of employment (full-time, part-time, and unemployed), as in other studies [20]. Although not statistically significant in the present study, the gradual decrease in the LTCRL scores was notable. A qualitative study reported that LTC professionals with abundant experience of conventional home care services faced more challenges to this new approach of caring for the elderly [32]. Hence, we recommend that in-service education for home care workers in Taiwan should be more focused on reablement in the future.

This study had several limitations. First, the pilot assessment for the scale recruited participants with purposive sampling. Although we recruited participants from northern, central, and southern Taiwan, this is not representative of the overall home care worker population in Taiwan. Second, each category has an unbalanced number of questionnaire items. One of the expert panel members suggested the merging of the categories of access/obtain (5 items) and understand (4 items), resulting in a relatively equal number of items to the categories of process/appraise (11 items) and apply/use (9 items). However, considering the original conceptual framework for the literacy definition [17], we decided to maintain four categories in the study. Third, the Delphi method was used to obtain data from expert consensus, and it is considered a validation process. However, given the dichotomy response design for the scale and a highly correct response rate in the pilot test, the indicators of construct validity were not as good as the criteria used in testing construct validity through confirmatory factor analysis. We recommend that in future studies, developing an assessment scale through the Delphi method should also include testing the enhanced validity, such as with construct validity. Fourth, the insufficient number of participants enrolled in the pilot study could be a bias in testing reliability and construct validity for the scale items, and it is suggested to include 5–10 participants per question item for testing the scale. The sample size for testing the self-developed questionnaires should be considered carefully in the future. The limitations of the study were mostly qualitative in nature, and the Delphi method is generally recognized as an effective tool for determining expert consensus when there is limited or no definitive evidence and measurement tools have not been developed [23].

## Conclusions

Reablement is a philosophy of change in LTC. Taiwan has officially introduced reablement programs to assist elderly individuals re-learn or regain their ability to perform ADLs independently [8]. This study is the first to develop a Chinese version of the LTCRL assessment scale in home care workers in Taiwan through a modified Delphi method. The findings suggest that in-service educational programs for home care workers in Taiwan should not only involve lectures and case reports but emphasize further on scenario-based exercises related to the advanced competencies of LTCRL.

## Supplementary Information

**Additional file 1.** Appendix. Complete list of categories and competencies with the final Likert score on the three-round Delphi.

## Data Availability

The datasets used and/or analyzed during the current study are available from the corresponding author on reasonable request.

## Abbreviations

LTC: Long-term care
LTCRL: Long-term care reablement literacy
ADL: Activities of daily living
