# Supplementary material for: Developing an assessment scale for long-term care reablement literacy in home care workers in Taiwan using a modified Delphi method
Source: BMC Geriatr. 2020 Nov 4;20:448. doi: 10.1186/s12877-020-01854-8 (PMC7640426; doi:10.1186/s12877-020-01854-8)
Supplement: Supplementary file 1 — Additional file 1. Appendix. Complete list of categories and competencies with the final Likert score on the three-round Delphi. [file 12877_2020_1854_MOESM1_ESM.docx]

**Appendix**

Complete list of categories and competencies with the final Likert score on the three-round Delphi.

| Competency category | Scenario-based questionnaire statement | Experts’ consensus scores on median (IQR) | | |
| --- | --- | --- | --- | --- |
|  |  | 1-round | 2-round | 3-round |
| Understand | 1-1 Hold for _____ seconds when performing a functional training task. | 4.00 (1.00) | 5.00 (1.00) | 5.00 (0.50) |
| Understand | 1-2 Keep breath _____ to prevent cardiopulmonary stress. | 5.00 (1.00) | 5.00 (1.00) | 5.00 (0.50) |
| Process/Appraise | 1-3 Repeat _____ times for each functional training by the prescription of therapist. | 4.00 (1.00) | 5.00 (1.00) | 5.00 (0.00) |
| Process/Appraise | 1-4 In sit-to-stand process, both feet keep _____ and the body lean _____. | 4.00 (0.50) | 4.50 (1.00) | 5.00 (0.50) |
| Understand | 1-5 Choose the best answer regarding accessory proper for walking exercise _____. | 4.50 (1.00) | 5.00 (0.25) | 5.00 (0.50) |
| Access/Obtain | 1-6 The role of _____ is to evaluate, design care plan, and follow up the reablement training programs after fracture surgery. | 4.00 (1.25) | 5.00 (1.00) | 5.00 (0.50) |
| Apply/Use | 2-1 Choose the best answer regarding functional training on ADL task. | 4.50 (1.00) | 5.00 (0.00) | 5.00 (0.00) |
| Apply/Use | 2-2 Choose the best answer regarding bladder training. | 4.00 (2.25) | 5.00 (1.00) | 5.00 (1.00) |
| Apply/Use | 2-3 _____ helps relieve caregiver’s burden when transferring. | 5.00 (1.00) | 5.00 (1.00) | 5.00 (1.00) |
| Process/Appraise | 2-4 Identify the wrong choice when transferring safely. | 4.00 (1.00) | 5.00 (0.25) | 5.00 (0.50) |
| Access/Obtain | 2-5 ____ is a place for consulting rehabilitation skills that help stroke patients regain functional performance. | 4.00 (1.25) | 4.50 (1.50) considered delete | Delete |
| Access/Obtain | 3-1 If you need an ergonomic wheelchair, _____ is a place to rent. | 4.50 (1.00) | 5.00 (1.00) | 5.00 (1.00) |
| Access/Obtain | 3-2 ____ is the person that helps evaluate environmental safety and assistive equipment. | 5.00 (1.00) | 5.00 (0.00) | 5.00 (0.00) |
| Process/Appraise | 3-3 Identify the contraindication that helps bed-ridden persons sit up. | 3.00 (2.00) | 4.50 (1.00) considered delete | Delete |
| Apply/Use | 3-4 The person’s normal heart rate is approximately 80 beat/min. If you help the person sit up and notice that the heart rate becomes _____ beat/min, you should lay down the person with careful observation. | 4.00 (2.00) | 4.50 (1.00) considered delete | Delete |
| Apply/Use | 3-5 The person’s normal blood pressure is approx. 140/90 mmHg. If you help the person sit up and notice that the blood pressure becomes _____mmHg, you should lay down the person with careful observation. | 3.50 (2.00) | 5.00 (1.00) | 4.00 (1.00) |
| Process/Appraise | 3-6 Choose the best answer regarding the proper time for bladder training. | 4.00 (2.00) | 4.00 (1.00) | 5.00 (1.00) |
| Apply/Use | 3-7 The recommended water supplement per day is 2000 ml. If a cup can accommodate 200 ml, the person is suggested to take ____ cups per day. | 4.00 (1.25) | 4.00 (1.00) | 5.00 (0.50) |
| Apply/Use | 3-8 Choose the best answer regarding bladder training. | 5.00 (1.00) | 5.00 (0.25) | 5.00 (0.50) |
| Apply/Use | 3-9 Setting up a hand rail in bathroom helps ______. | 4.00 (1.00) | 5.00 (0.25) | 5.00 (0.50) |
| Apply/Use | 3-10 The best place for home care electric bed is _____. | 4.00 (1.05) | 5.00 (1.00) | 5.00 (0.00) |
| Access/Obtain | 3-11 _____ is a document that contains information on personal training programs on ADL task designed by a therapist. | 5.00 (1.00) | 5.00 (0.25) | 5.00 (1.00) |
| Process/Appraise | 4-1 Repositioning bedfast persons at least every _____ hours is recommended. | 4.00 (1.50) | 4.50 (1.00) | 5.00 (0.50) |
| Understand | 4-2 The purpose for using air flotation bed is _____. | 4.00 (1.25) | 5.00 (0.25) | 5.00 (0.00) |
| Process/Appraise | 4-3 Which is the best answer regarding the prevention of pressure injuries in a reablement manner. | 4.00 (1.00) | 4.50 (1.00) | 5.00 (0.00) |
| Process/Appraise | 4-4 In positioning the accessory, bony prominence such as ______ should be avoided. | 4.00 (1.00) | 5.00 (1.00) | 5.00 (0.00) |
| Process/Appraise | 4-5 A persistently red, painful skin indicates pressure injury stage _____. | 4.00 (1.50) | 4.50 (1.00) | 5.00 (0.00) |
| Access/Obtain | 4-6 Reablement is an interdisciplinary team approach. When it comes to malnutrition, ______ may be the best professional you can consult. | 4.50 (1.25) | 5.00 (1.00) | 5.00 (0.00) |
| Process/Appraise | 5-1 Choose the reason for avoiding prolonged bed rest in older people. | 4.00 (1.00) | 5.00 (0.00) | 5.00 (1.00) |
| Process/Appraise | 5-2 After stroke, the case should _____ to prevent further deterioration in a reablement manner. | 4.00 (1.00) | 5.00 (1.25) | 5.00 (0.00) |
| Apply/Use | 5-3 The caregiver may _____ with the older people in order to reconstruct daily tasks. | 4.00 (1.00) | 4.00 (1.25) | 5.00 (1.00) |
| Process/Appraise | 5-4 An older person spends 6 hours in participating activities in adult day care center. What is the best practice in a reablement manner when the person returns home? | 4.00 (1.00) | 5.00 (1.00) | 5.00 (0.00) |
| Process/Appraise | 5-5 The vital sign (_____) can indicate that siting on the chair for 3 hours can be all right as a home exercise. | 4.00 (2.00) considered delete | delete | delete |

IQR: interquartile range
